# Sarcoidosis With Bilateral Testicular Involvement Resembling Testicular Cancer: A Rare Case Report

**DOI:** 10.7759/cureus.23982

**Published:** 2022-04-09

**Authors:** Taner Hacıosmanoğlu, Semih Türk, İbrahim H Baloğlu, Eminegül Yavuzsan, Abdullah H Yavuzsan

**Affiliations:** 1 Urology, Sisli Hamidiye Etfal Training and Research Hospital, University of Health Sciences, Istanbul, TUR; 2 Pulmonology and Critical Care, Yedikule Chest Diseases and Thoracic Surgery Training and Research Hospital, İstanbul, TUR

**Keywords:** steroid treatment, testicular biopsy, testicular cancer, testicular sarcoidosis, sarcoidosis

## Abstract

Sarcoidosis is a granulomatous inflammatory disease that could potentially involve multiple organ systems. It causes noncaseating granulomas in tissues, and at least two organs must be involved to make a diagnosis. In sarcoidosis patients, if there is a mass in the testicles, a testicular biopsy should be performed to exclude malignancies because of infrequent testicular involvement. We present a 23-year-old male diagnosed with sarcoidosis who had a bilateral testicular mass. A testicular biopsy was performed because of bilateral involvement. The biopsy revealed a diagnosis of sarcoidosis. After high-dose steroid treatment, the lesions regressed. This paper presents a sarcoidosis case with testicular involvement that imitates testicular tumors. Testicular tumors and testicular involvement of sarcoidosis are two different pathologies that may mimic each other, confuse clinicians, and/or lead to misdiagnosis and mistreatment.

## Introduction

Sarcoidosis is a granulomatous inflammatory disease that could potentially involve multiple organ systems and causes non-caseating granulomas in tissues [1]. Although the lung is the most commonly affected organ, this disease can involve almost any organ in the body [2,3]. The liver, skin, eyes, and spleen are the other organs frequently affected, while the breast, ovary, stomach, and testes are seldom involved [1,4-7]. In a patient with sarcoidosis, if a mass is found in the testicles, a testicular biopsy should be done to exclude malignancies because of infrequent testicular involvement of sarcoidosis [7]. Therefore, if the testes are affected, the disease might resemble testicular tumors. With this condition, clinicians should consider testicular tumors in their differential diagnosis. In the literature, there are few case reports concerning testicular involvement in sarcoidosis. Accordingly, this paper presents a sarcoidosis case with testicular involvement that imitated a testicular tumor and regressed after steroid treatment.

## Case presentation

**Figure 1:**
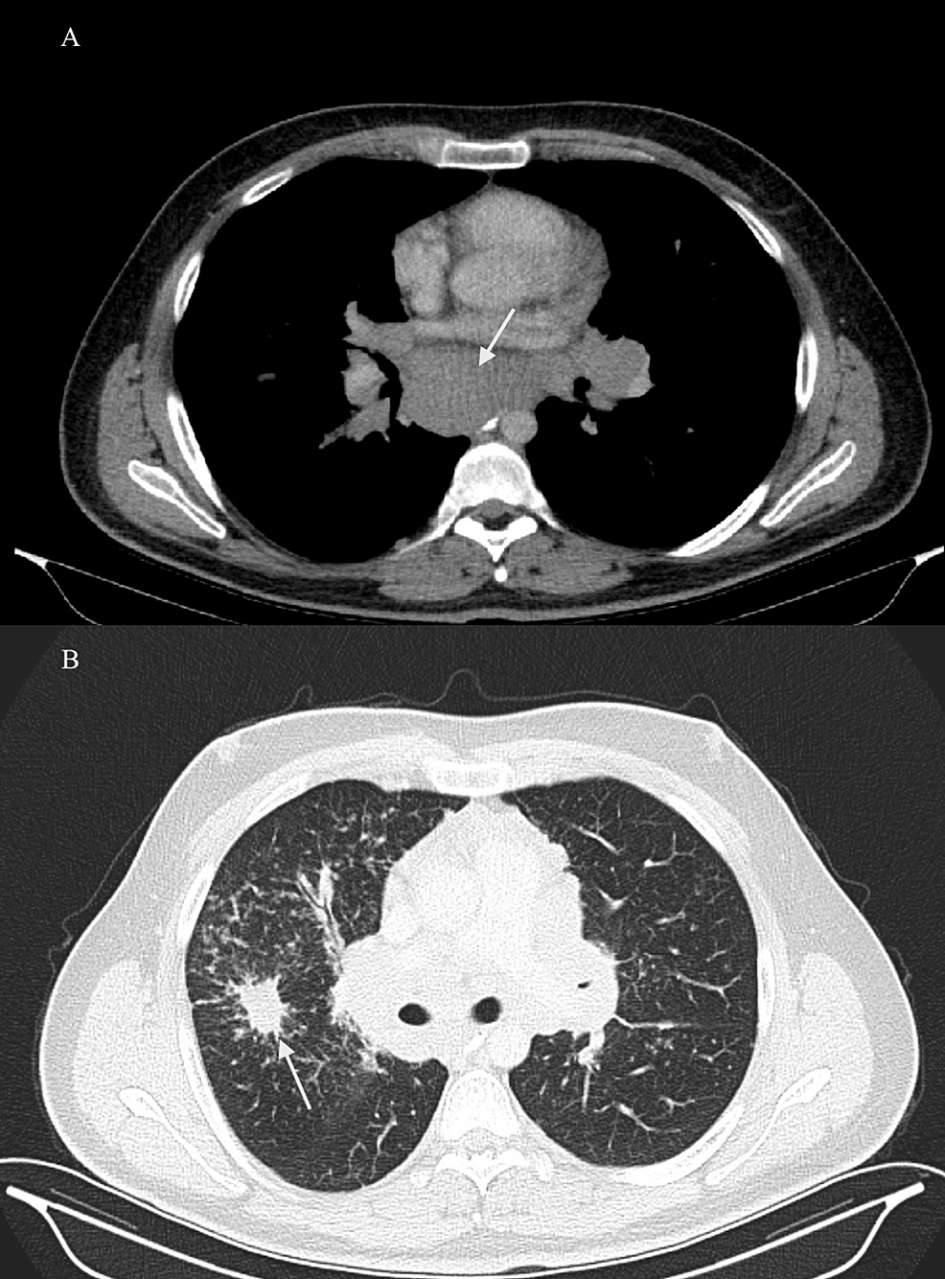
Thoracal CT shows the biggest lymph node (5x4 cm) in the mediastinum (A) and a nodule in the upper right lobe of the right lung (B) with arrows.

A 23-year-old male patient with known asthma and a sarcoidosis history came to our outpatient urology clinic with a complaint of left testicular pain. He had been previously diagnosed with sarcoidosis by transbronchial biopsy and mediastinoscopy and had received steroid treatment six months before coming to our clinic. Upon physical examination, left testicular tenderness was detected. Scrotal Doppler ultrasonography (USG) exposed multiple hypoechoic lesions with irregular borders in the left testicle, with the largest being 6 × 6 mm. The USG also showed a regularly bordered hypoechoic 2 mm lesion in the right testicle. The laboratory analysis showed that the patient’s ß-human chorionic gonadotropin (β-HCG) and alpha-fetoprotein (AFP) levels were normal, and his lactate dehydrogenase (LDH) level was measured as 274 U/L. Contrast-enhanced thoracoabdominal computed tomography (CT) documented multiple conglomerated lymphadenopathies (LAPs) in every lymphatic station in the mediastinum, with the largest lymph node’s size being 5 × 4 cm (Figure 1A). Multiple nodules were detected in the parenchyma of both lungs and the upper lobe of the right lung. The size of the nodule in the upper lobe of the right lung was 2.5 cm (Figure 1B).

**Figure 2:**
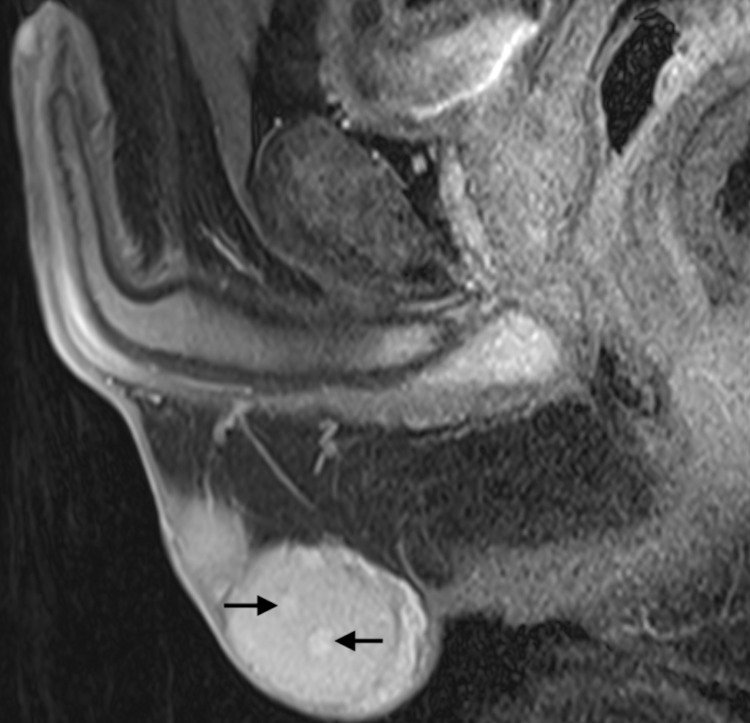
T2-weighted MRI shows multiple nodular lesions below 6 mm in size, with weak heterogeneous contrast uptake with arrows.

Because the patient had a diagnosis of sarcoidosis and bilateral testicular involvement, we evaluated the patient via contrast-enhanced abdominopelvic magnetic resonance imaging (MRI), which showed multiple nodular lesions smaller than 6 mm in size with weak heterogeneous contrast uptake (Figure 2).

**Figure 3:**
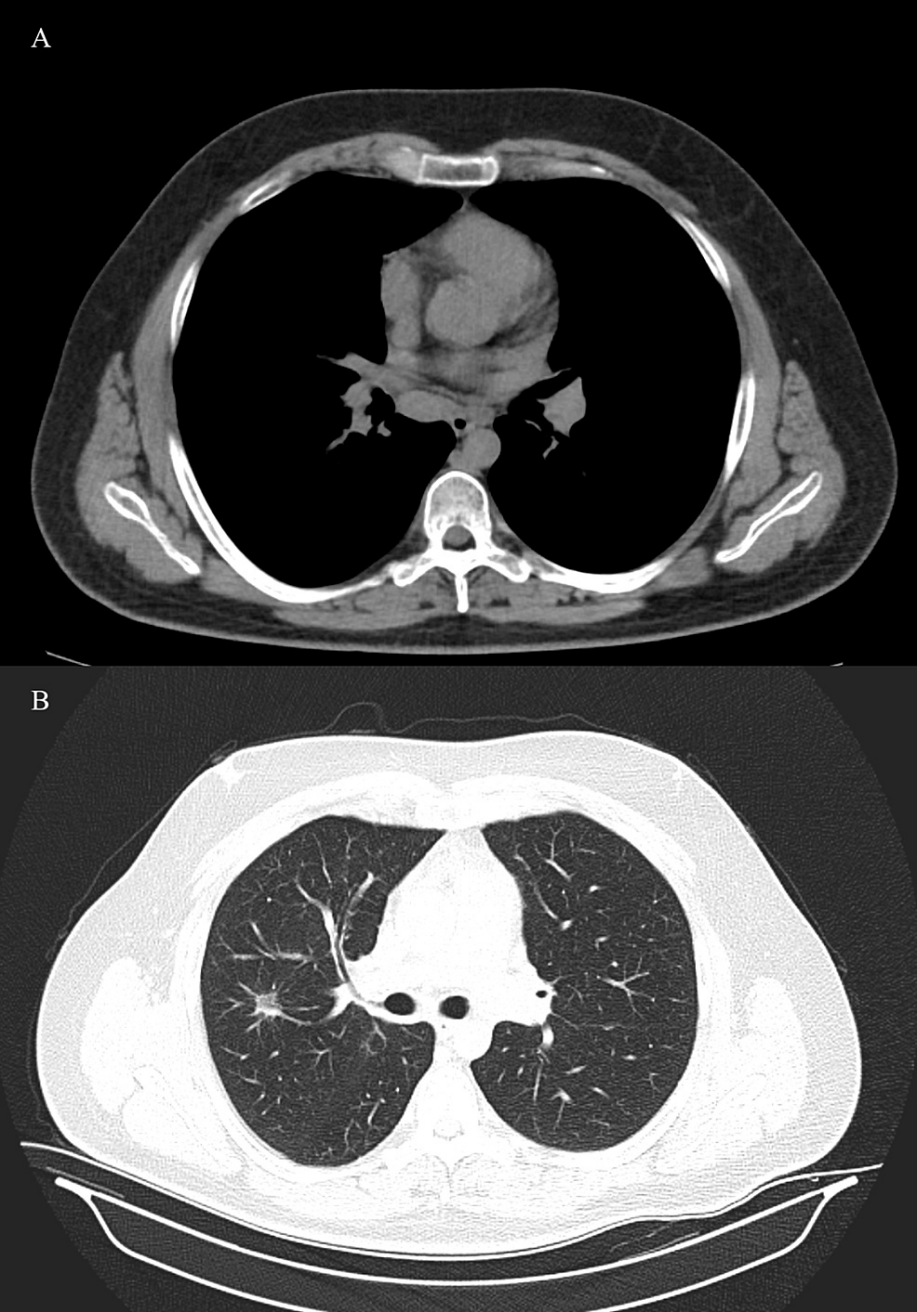
Thoracal CT shows the regression in the size of the nodules in the lungs (A) and the mediastinal lymphadenopathies (B) after high-dose steroid treatment.

Afterward, we recommended radical orchiectomy or testicular biopsy because of his sarcoidosis diagnosis, bilateral testicular involvement, and desire to preserve fertility. We also recommended a spermiogram to evaluate the patient’s fertility state, given that sarcoidosis and testicular tumors might lead to infertility, which the patient refused. He decided to undergo a testicular biopsy. Under spinal anesthesia, the left testicle was exposed through a left scrotal horizontal incision. A USG-assisted needle biopsy was performed on the lesion. Frozen section analysis of the biopsy specimen showed non-necrotizing granulomatous findings in the testicle. No testicular tumor cells were seen. Therefore, we referred the patient to the pulmonology department for treatment. In accordance with the recommendations of the pulmonologists, he started to receive high-dose steroid treatment by getting 32 milligrams of methylprednisolone per day, and the therapy terminated in a dose diminishing way within six months. After receiving high-dose steroid treatment for six months, the patient visited our outpatient clinic for control, as we suggested. Afterward, we checked the testicular tumor marker levels again, and his LDH, AFP, and β-HCG levels were all normal. In the control USG, testicular parenchyma was homogenous and isoechoic, and there were no solid lesions on either side. In non-contrast thoracal CT, the parenchymal nodules were dramatically regressed, and mediastinal LAPs were smaller in size (Figures 3A-3B).

**Figure 4:**
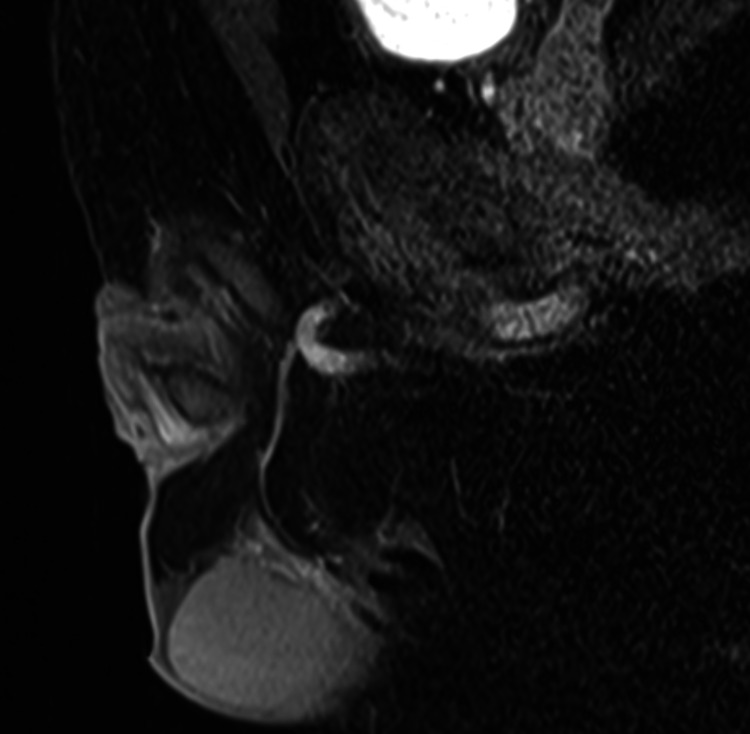
T2-weighted MRI shows no abnormal findings in the left testicle after treatment.

There were no abnormal findings in the MRI, including LAPs and solid lesions (Figure 4).

## Discussion

Sarcoidosis is a multisystemic disease characterized by non-caseating granulomas. Urogenital sarcoidosis is quite rare, and the urogenital system is affected in only 0.2% of all cases [8]. In a published series of 60 cases, the most common involvement in the urogenital tract was in the epididymis (73%), followed by the testis (47%), spermatic cord (8%), and prostate (3%) [9]. Patients with testicular sarcoidosis generally go to outpatient clinics with a unilateral, painless nodular mass in the testis [10]. Notably, there have been reports of infertility due to testicular sarcoidosis [11]. Intrathoracic findings are present in more than 80% of patients with urogenital sarcoidosis [12]. In our case, it was known that the patient had thoracic involvement and a sarcoidosis diagnosis. Bilateral testicular involvement was also seen in this patient, which is an extremely rare condition.

For differential diagnosis, tuberculosis, fungal infections, and granulomatous diseases such as berylliosis must be excluded, and testicular cancer should be kept in mind [13]. Testicular carcinoma is strongly related to sarcoidosis; the risk of testicular cancer in young white men with sarcoidosis is approximately 100 times greater than that of the general population [14]. These patients must be followed up regularly over a long period due to the high risk of cancer. Apart from this, it has been reported that testicular sarcoidosis spontaneously regresses by approximately 80% within two years [12]. Serum tumor markers (AFP, ß-HCG, LDH) can be utilized to differentiate sarcoidosis and germ cell testicular carcinoma; there is no apparent relationship between sarcoidosis and serum levels of AFP and ß-HCG. Moreover, it has been reported that the LDH level can be increased in testicular sarcoidosis, just as it was in our case [15].

The effects of sarcoidosis on fertility have not yet been illuminated. The development of obstructive granuloma and fibrosis secondary to epididymal involvement may lead to azoospermia. Infertility has been reported due to sarcoidosis in some cases [11]. Oligospermia and azoospermia may be fixed by corticosteroid treatment, leading to a decrease in epididymal obstruction [16]. In our case, there is no epididymal involvement; however, we did not have any information about the patient’s fertility because the patient refused a semen analysis. Thus, we cannot interpret the contribution of corticosteroid treatment to the patient’s fertility.

There is no consensus on the management of testicular sarcoidosis in the literature. The general opinion among the authors is radical orchiectomy, especially in patients with unilateral masses due to the increased risk of malignancy. However, if sarcoidosis is suspected and tumor markers are negative in patients with bilateral involvement, the biopsy is preferred [17]. Excisional biopsy or needle biopsy may be preferred. A biopsy may be a more appropriate approach for avoiding radical procedures, especially in patients with suspected sarcoidosis who do not have risk factors for testicular cancer and whose markers are negative. Bilateral testicular involvement, the desire to protect fertility, the negativity of tumor markers, and the diagnosis of sarcoidosis led us to perform a testicular biopsy in this case.

## Conclusions

Testicular sarcoidosis should be considered in patients with known sarcoidosis histories applying with testicular mass. Testicular biopsy or radical orchiectomy options may be offered to the patient. Moreover, because there is an increased risk of testicular cancer in patients with sarcoidosis, urologists should regularly examine them for testicular tumors.
